# The impact of diabetes and subclinical hypothyroidism association with coronary artery calcium: results from the Brazilian Longitudinal Study of Adult Health (ELSA-Brasil)

**DOI:** 10.20945/2359-4292-2022-0375

**Published:** 2023-12-01

**Authors:** Aída de Melo Spilack, Alessandra C. Goulart, Carolina C. P. S. Janovsky, Bianca de Almeida-Pittito, Paulo A. Lotufo, Márcio Sommer Bittencourt, Giuliano Generoso, Itamar de Souza Santos, Isabela M. Bensenor

**Affiliations:** 1 Universidade de São Paulo Faculdade de Medicina São Paulo SP Brasil Faculdade de Medicina, Universidade de São Paulo, São Paulo, SP, Brasil; 2 Universidade de São Paulo Hospital Universitário Centro de Pesquisa Clínica e Epidemiológica São Paulo SP Brasil Centro de Pesquisa Clínica e Epidemiológica, Hospital Universitário, Universidade de São Paulo, São Paulo, SP, Brasil; 3 Universidade Federal de São Paulo Faculdade de Medicina Departamento de Medicina Preventiva São Paulo SP Brasil Departamento de Medicina Preventiva, Faculdade de Medicina, Universidade Federal de São Paulo, São Paulo, SP, Brasil; 4 University of Pittsburgh Department of Medicine and Department of Radiology Pittsburgh PA USA Department of Medicine and Department of Radiology, University of Pittsburgh, Pittsburgh, USA

**Keywords:** Coronary artery calcium, diabetes mellitus, subclinical hypothyroidism, subclinical atherosclerosis, cardiovascular disease

## Abstract

**Objective:**

We aimed to analyze the association of diabetes and subclinical hypothyroidism with subclinical atherosclerosis measured by coronary artery calcium (CAC) in the baseline of the ELSA-Brasil study.

**Materials and methods:**

CAC was measured using a 64-detector computed tomographic scanner. The association of CAC > 0 was presented as an odds ratio (OR) and 95% confidence intervals (95%CI) in logistic models and as β (95%CI) in linear models after multivariable adjustment for confounders.

**Results:**

We analyzed 3,809 participants (mean-age (SD) 50.5 (8.8); 51.7% women). In the main analysis, we did not find an association of diabetes and subclinical hypothyroidism with CAC. However, in stratified analysis according to age strata, we found no significative interaction terms, an important heterogeneity between the groups, with the younger age strata showing an association of the group with both diseases and CAC > 0 (OR 7.16; 95%CI, 1.14; 44.89) with a wide but significative 95%CI, suggesting that the smaller number of participants in the younger group may influence the results. Our findings also showed an association of CAC > 0 and log (CAC+1) with diabetes in logistic (OR, 1.31; 95%CI, 1.05-1.63) and linear models (β, 0.24, 0.16, 0.40), respectively. Diabetes was independently associated with CAC > 0 in linear models.

**Discussion:**

In conclusion, our results showed a great heterogeneity in stratified analysis based on age in the younger age strata. Although we found no significant interaction factors, the smaller sample size for the analysis may influence the negative findings.

## INTRODUCTION

The International Diabetes Federation Atlas reported that 537 million adults around the world are living with diabetes, a 16% increase since the previous IDF estimates in 2019 (1). However, some data suggest that these numbers are underestimated and the real numbers may be worse in the following years (2). Thyroid dysfunction is also very frequent, with a high prevalence worldwide, especially considering subclinical thyroid diseases (3,4). Subclinical thyroid diseases are less symptomatic, and a great majority of patients remain undiagnosed in clinical practice (5).

Several authors have recognized the possible association of diabetes and subclinical hypothyroidism (6,7). Some studies have shown that patients with type 2 diabetes and subclinical hypothyroidism are more likely to have microvascular complications, such as diabetic nephropathy (8–10), retinopathy (11) and peripheral neuropathy, than individuals with only diabetes (12). However, these findings were not confirmed in other studies (13–15).

Researchers have conducted few studies to evaluate the synergic effect of diabetes and subclinical hypothyroidism with macrovascular complications of diabetes, such as coronary heart disease (7,9). Han and cols. (2015), in a systematic review and meta-analysis, reported a non-significant association of diabetes and subclinical hypothyroidism with coronary heart disease [OR of 1.59, 95%CI, 0.92-2.76] (7). Contrasting with these findings, Jia and cols. (2015) reported an association of diabetes and subclinical hypothyroidism with coronary heart disease (9). Therefore, there is conflicting data about the possible synergic association of diabetes and subclinical hypothyroidism in microvascular and macrovascular complications of diabetes in individuals with both diseases.

Coronary artery calcium (CAC) scores are a surrogate marker of subclinical atherosclerosis and a predictor of future cardiovascular events (16,17). Although some studies showed an association of diabetes with CAC (18,19), subclinical hypothyroidism with CAC (20) or even high-normal TSH levels with CAC (21), researchers have conducted few other studies to analyze the associations among subclinical hypothyroidism, diabetes and CAC. Posadas-Romero and cols. (2014) reported an association of subclinical hypothyroidism, metabolic syndrome and its components with subclinical atherosclerosis measured by CAC and fat liver disease (22). In addition, a cross-sectional study in Brazil reported a strong positive association between CAC > 100 and subclinical hypothyroidism in older men with a Framingham risk score ≥ 10% and having diabetes as one of the cardiovascular risk factors evaluated (23). Recently an association with higher CAC score values was reported in patients in hemodialysis, high TSH levels and a high prevalence of diabetes (24). Subclinical hypothyroidism in high-risk groups may represent an additional risk factor for coronary artery calcification in individuals with intermediate and high cardiovascular risk scores. It is important to note that all these studies showed possible associations among subclinical hypothyroidism, diabetes and CAC in subgroup analyses.

We aimed to analyze the association of diabetes, subclinical hypothyroidism or both diseases with subclinical atherosclerosis measured by CAC using data from the baseline examination of the Brazilian Longitudinal Study of Adult Health (ELSA-Brasil). Our hypothesis is that the subgroup of subjects with diabetes and subclinical hypothyroidism would be associated with higher CAC scores compared to other subgroups with only diabetes or subclinical hypothyroidism, using the group of participants without diabetes and subclinical hypothyroidism as the reference.

## MATERIALS AND METHODS

The ELSA-Brasil is a prospective cohort study that enrolled 15,105 civil servants age 35 to 74 from five public universities and research institutes located in 6 state capitals: Salvador (BA), Belo Horizonte (MG), Vitória (ES), Rio de Janeiro (RJ), São Paulo (SP) and Porto Alegre (RS) (25–27). Inclusion criteria are being a 35-to 74-year-old active or retired employee of one of the six institutions. Exclusion criteria included not having severe communication or cognitive problems; the near-future possibility of stopping working in the institutions soon after the enrollment in the study and intention to move to neighborhoods outside the metropolitan area in which the institution was localized, compromising participation in the study and being pregnant (25). The study was conducted in accordance with the Declaration of Helsinki and approved by the Ethic Board of all six centers. All participants provided an informed consent.

**Figure 1:**
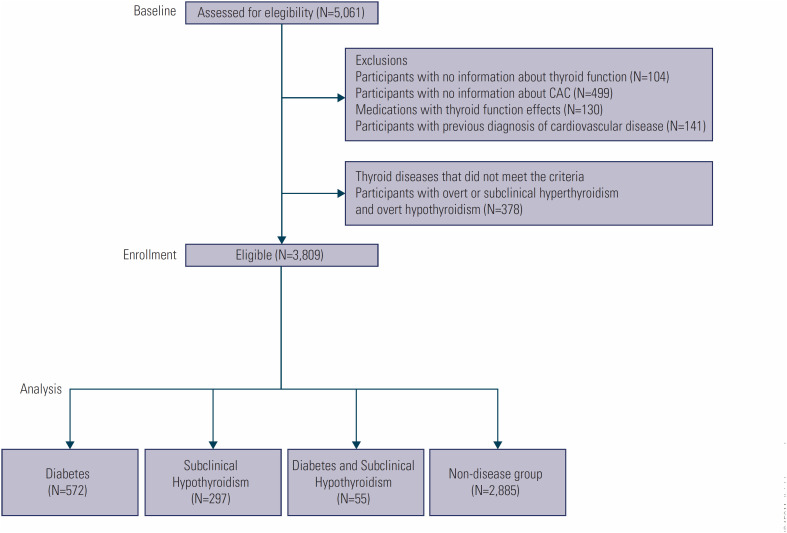
Description of the sample included in the analysis.

This is a cross-sectional analysis using data from the baseline of the ELSA-Brasil conducted between 2008-2010, and it was registered with Brazil Platform with a CAAE number of 42178221.8.0000.0076. The present analysis is a cross-sectional study using a subsample of participants from ELSA-Brasil of the research center of São Paulo (N = 5,061). From those, we excluded 104 participants with no data regarding thyroid diseases and 499 without information about CAC at baseline, 130 using drugs that may alter thyroid function and 141 with previous cardiovascular diseases. We also excluded 378 participants with overt or subclinical hyperthyroidism and overt hypothyroidism, leaving 3,809 participants for this analysis: 2,885 with no disease, 297 with only subclinical hypothyroidism, 572 with only diabetes and 55 with both diseases (Figure 1).

### Definition of diabetes

The definition of diabetes included a self-reported medical diagnosis of diabetes, use of drugs to treat diabetes, fasting plasma glucose levels ≥ 7.0 mmol/L, 2-h glucose levels ≥ 11.1 mmol/L or HbA1c ≥ 6.5% (28).

A 12-hour fasting blood sample was drawn in the morning following study procedures (28–30). A standardized 75-g oral glucose tolerance test (OGTT) was conducted with all participants without a previous diagnosis of diabetes based on the above criteria. Glucose levels were measured following the hexokinase method (ADVIA Chemistry; Siemens, Deerfield, Illinois), and HbA1c was measured using high pressure liquid chromatography (Bio-Rad Laboratories, Hercules, California).

### Thyroid function

TSH (normal range: 0.4-4.0 mIU/L), FT4 (0.93-1.7 ng/dL) and FT3 (0.20-0.44 ng/dL) were determined using a third-generation immunoenzymatic assay (Roche Diagnostic, Manheim, Germany) (28). The analysis included euthyroid participants (TSH levels 0.4-4.0 mIU/L with no use of levothyroxine/anti-thyroid drugs) and subclinical hypothyroidism (TSH > 4.0 mIU/L with FT4 0.93-1.7 ng/dL). We excluded participants with overt thyroid diseases, with subclinical hyperthyroidism or using medication that alter thyroid function (amiodarone, carbamazepine, carbidopa, furosemide, haloperidol, heparin, levodopa, lithium, metoclopramide, phenytoin, propranolol, primidone, rifampicin, steroids and valproic acid) (Figure 1) (31,32).

### Measurement of coronary artery calcium (CAC)

All participants underwent a CAC examination performed with a 64-detector computed tomographic scanner (Brilliance 64; Philips Healthcare, Best, The Netherlands). After the scout images were taken, each patient underwent an electrocardiogram-gated prospective calcium score examination with a tube potential of 120 kV and a tube current adjusted to body habitus. Images were reconstructed in 2.5-mm-thick slices using standard filtered back projection. The CAC was expressed in Agatston units, and an experienced cardiologist evaluated the percentile in a blinded fashion using semiautomatic software (Calcium Scoring, Philips Workstation). CAC severity was further categorized according to an Agatston score of 0 or >0 (33–35) or log transformed (log CAC+1).

### Other variables

We evaluated sociodemographic characteristics, such as sex, age as continuous and stratified (35-44; 45-54; 55-64; 65-74 strata), educational attainment (less than high school, high school and some college and at least complete college), mean average family monthly income (≤US$ 1245, US$ 1246 to US$ 3319 and ≥ US$ 3320) and self-reported race/skin color (White, Mixed, Black, Asian and Indigenous). BMI was stratified as < 30 kg/m^2^ and ≥ 30 kg/m^2^. Smoking and alcohol use were categorized as never, past or current. Blood pressure (BP) was measured using a validated Omron HEM 705CPINT oscillometric device. Three blood pressure measurements were taken at 1-minute intervals, and the average of the last two measurements was considered the value for casual systolic and diastolic blood pressure. The definition of hypertension was based on current use of medication to treat hypertension or systolic blood pressure ≥ 140 mmHg, and/or diastolic blood pressure ≥ 90 mmHg . The definition of dyslipidemia was based on LDL-cholesterol levels > 130 mg/dL or the use of any type of lipid-lowering medication. Leisure time physical activity was classified according to the World Health Organization criteria using the long version of the International Physical Activity Questionnaire (IPAQ), in which being physically active meant at least 150 min of moderate-intensity, 75 min of high-intensity leisure-time aerobic physical activity or the combined equivalent of both each week. Any weekly activity below the previous threshold was classified as partly active, and the remaining participants were classified as inactive (36).

### Statistical analysis

Categorical variables are expressed as absolute numbers with the respective frequencies and analyzed using the chi-square test. Continuous variables are presented as mean (standard deviations) and compared using ANOVA of normal distributions, and they are presented as medians (interquartile range) and analyzed using nonparametric tests if they show non-normal distribution.

Logistic regressions models were built to evaluate the association between diabetes, subclinical hypothyroidism and both diseases combined as independent variables and CAC > 0 Agatston units as the dependent variable considered in all models. ORs are presented without adjustment and after adjustment for sociodemographic variables (age, sex, self-reported race and education – Model 1), and multivariable adjustment for all variables included in Model 1, more smoking, alcohol intake and dyslipidemia (Model 2). Linear regression models using log (CAC+1) were presented as β coefficients (95%CI) using the same multivariable adjustments of the logistic models. We performed some sensitivity analyses according to age strata, excluding obese individuals (BMI ≥ 30 kg/m^2^), and sex. The interaction between diabetes and subclinical hypothyroidism was tested using interaction terms in Model 2 of the logistic and linear regression models.

We considered p values < 0.05 significant. Data were analyzed using the Statistical Packages for Social Sciences SPSS version 25.0.

## RESULTS

Table 1 shows baseline characteristics according to the presence of diabetes, subclinical hypothyroidism, both conditions and no diseases (reference group). Age, BMI and WC are higher in individuals with both diseases than in the other subgroups. The frequency of women was lower in the subgroup of participants with diabetes than in the other subgroups. Frequencies of Whites and participants who completed college or more were lower in the group of patients with only diabetes than in the other subgroups (P < 0.0001). Current smoking was less common in participants with subclinical hypothyroidism (P < 0.0001). The frequency of some cardiovascular risk factors, such as hypertension and dyslipidemia, were higher in the group of patients with diabetes and both diseases. Participants with diabetes presented a higher frequency of CAC > 0 than the other subgroups.

**Table 1:** General and clinical characteristics of the sample according to the presence or not of subclinical hypothyroidism or diabetes

		Diagnosis of diabetes or subclinical hypothyroidism			
		No	Only subclinical hypothyroidism	Only diabetes	Both
		N = 2,885	N = 297	N = 572	N = 55
Age (years)		49.5 (49.2-49.9)	51.0 (50.0-52.0)	54.4 (53.7-55.1)	54.5 (52.0-57.0)
Women (%)		1,548 53.7 (51.8-55.5)	155 52.2 (46.3-58.0)	233 40.7 (36.7-44.9)	35 63.6 (49.5-75.9)
Self-reported race (%)					
	White	1,677 58.8 (57.0-60.7)	187 63.2 (57.4-68.6)	276 49.1 (44.9-53.3)	33 62.3 (47.9-74.9)
	Mixed	632 22.2 (20.7-23.8)	66 22.3 (17.8-27.6)	135 24.0 (20.6-27.8)	10 18.9 (9.9-32.4)
	Black	399 14.0 (12.8-15.3)	29 9.8 (6.8-13.9)	102 18.1 (15.1-21.6)	7 13.2 (5.9-26.0)
	Asian	112 3.9 (3.3-4.7)	10 3.4 (1.7-6.3)	43 7.7 (5.7-10.2)	2 3.8 (0.7-14.1)
	Indigenous	30 1.1 (0.7-1.5)	4 1.4 (0.4-3.7)	6 1.1 (0.4-2.4)	1 1.9 (0.1-11.4)
Education					
	Less than high-school	384 13.3 (12.1-14.6)	49 16.5 (12.6-21.3)	136 23.8 (20.4-27.5)	13 23.6 (13.7-37.3)
	High-school and some college	1,231 42.7 (40.9-44.5)	116 39.1 (33.5-44.9)	228 39.9 (35.8-44.0)	24 43.6 (30.6-57.6)
	Complete college or more	1,270 44.0 (42.2-45.9)	132 44.4 (38.7-50.3)	208 36.4 (32.4-40.5)	18 32.7 (21.0-46.8)
Body mass index (BMI) kg/m^2^		26.7 (26.5-26.9)	27.5 (26.9-28.1)	29.5 (29.1-29.9)	30.6 (28.9-32.3)
Waist measurement (cm)		88.3 (87.9-88.7)	89.8 (88.4-91.2)	96.8 (95.8-97.9)	97.3 (93.4-101.2)
Hypertension (%)		676 23.4 (21.9-25.0)	77 25.9 (21.1-31.4)	329 57.5 (53.3-61.6)	31 56.4 (42.4-69.4)
Dyslipidemia (%)		1,156 40.1 (38.3-41.9)	113 38.0 (32.5-43.9)	299 52.3 (48.1-56.4)	27 49.1 (35.5-62.8)
Smoking (%)					
	Never	1,559 54.0 (52.2-55.9)	170 57.2 (51.4-62.9)	279 48.8 (44.6-53.0)	34 61.8 (47.7-74.3)
	Past	827 28.7 (27.0-30.4)	105 35.4 (30.0-41.1)	197 34.4 (30.6-38.5)	15 27.3 (16.5-41.2)
	Current	499 17.3 (15.9-18.7)	22 7.4 (4.8-11.2)	96 16.8 (13.9-20.2)	6 10.9 (4.5-22.9)
Alcohol use (%)					
	Never	332 11.5 (10.4-12.7)	38 12.8 (9.3-17.3)	69 12.1 (9.6-15.1)	10 18.2 (9.5-31.4)
	Past	538 18.6 (17.3-20.1)	54 18.2 (14.1-23.1)	122 21.3 (18.1-25.0)	11 20.0 (10.9-33.4)
	Current	2,015 69.8 (68.1-71.5)	205 69.0 (63.4-74.2)	381 66.6 (62.6-70.4)	34 61.8 (47.7-74.3)
Physical activity (%)					
	Inactive	2,184 78.7 (77.1-80.2)	214 75.6 (70.1-80.4)	447 79.7 (76.1-82.9)	41 80.4 (66.5-89.7)
	Insufficiently active	364 13.1 (11.9-14.4)	40 14.1 (10.4-18.9)	68 12.1 (9.6-15.2)	8 15.7 (7.5-29.1)
	Active	228 8.2 (7.2-9.3)	29 10.2 (7.1-14.5)	46 8.2 (6.1-10.9)	2 3.9 (0.7-14.6)
CAC > 0		273 19.3 (17.3-21.5)	23 14.8 (9.8-21.6)	62 33.3 (26.7-40.7)	7 26.9 (12.4-48.1)
CAC (Agatston) median (IQR)		0 (0-0)	0 (0-1)	0 (0-56)	0 (0-26)

IQR: interquartile range.

Table 2 presents the ORs for the associations between subclinical hypothyroidism, diabetes and both diseases with CAC according to age strata and in the entire sample.

**Table 2:** Logistic models (Odds ratio [OR] and 95% Confidence Interval [95%CI]) and linear regression models (β coefficient [β] (95%CI) for the association of subclinical hypothyroidism, diabetes and both diseases with CAC > 0 according to age-strata and the entire sample

Age-strata 35 to 44 years N =1009	Crude	Model 1	Model 2
Logistic regression models	OR (95%CI)	OR (95%CI)	OR (95%CI)
CAC > 0			
Subclinical hypothyroidism	1.74 (0.79; 3.79)	1.82 (0.80; 4.13)	1.73 (0.72; 4.15)
Only diabetes	4.41 (2.40; 8.10)	3.40 (1.78; 6.52)	2.96 (1.43; 6.14)
Both diseases	5.81 (1.10; 30.64)	4.54 (0.78; 26.61)	7.16 (1.14; 44.89)
Linear regression models			
Log CAC + 1	β (95%CI)	β (95%CI)	β (95%CI)
Subclinical hypothyroidism	0.17 (-0.012; 0.47)	0.14 (-0,14; 0,43)	0.19 (-0,11; 0,49)
Only diabetes	0.26 (0.17; 0.36)*	0.23 (0.14; 0.33)*	0.22 (0.12; 0.31)*
Both diseases	0.04 (-0,05; 0.14)	0.04 (-0.05; 0.13)	0.03 (-0.06; 0.12)
**Age-strata 45 to 54 years N = 1636**	**Crude**	**Model 1**	**Model 2**
Logistic regression models	OR (95%CI)	OR (95%CI)	OR (95%CI)
CAC > 0			
Subclinical hypothyroidism	0.93 (0.59; 1.49)	0.82 (0.50; 1.34)	0.81 (0.49; 1.36)
Only diabetes	1.74 (1.28; −2.37)	1.44 (1.03; 2.01)	1.07 (0.75; 1.52)
Both diseases	0.63 (0.18; 2.13)	0.49 (0.14; 1.77)	0.26 (0.06; 1.18)
Linear regression models			
Log CAC + 1	β (95%CI)	β (95%CI)	β (95%CI)
Subclinical hypothyroidism	0.04 (-0.33; 0.26)	-0.06 (-0.35; 0.22)	-0.17 (-0.45; 0.12)
Only diabetes	0.22 (0.12; 0.31)*	0.16 (0.07; 0.26)*	0.08 (-0.02; 0.18)*
Both diseases	-0.001 (-0.13; 0.13)	-0.02 (-0.15; 0.10)	-0.04 (-0.17; 0.08)
**Age-strata 55 to 64 years N = 866**	**Crude**	**Model 1**	**Model 2**
Logistic regression models	OR (95%CI)	OR (95%CI)	OR (95%CI)
CAC > 0			
Subclinical hypothyroidism	0.98 (0.60; 1.60)	1.11 (0.66; 1.87)	1.08 (0.63; 1.85)
Only diabetes	1.94 (1.38; 2.74)	1.87 (1.28; 2.74)	1.48 (1.00; 2.19)
Both diseases	0.69 (0.25; 1.93)	0.96 (0.32; 2.93)	0.94 (0.29; 3.03)
Linear regression models			
Log CAC+1	β (95%CI)	β (95%CI)	β (95%CI)
Subclinical hypothyroidism	-0.09 (-0.58; 0.40)	-0.11 (-0.38; 0.22)	-0.00 (-0.51; 0.51)
Only diabetes	0.39 (0.22; 0.55)*	0.34 (0.18; 0.50)*	0.23 (0.02; 0.39)*
Both diseases	0.07 (-0.17; 0.31)	0.14 (-0.09; 0.37)	-0.14 (-0.09; 0.37)
**Age-strata 65 to 74 years N=298**	**Crude**	**Model 1**	**Model 2**
Logistic regression models	OR (95%CI)	OR (95%CI)	OR (95%CI)
CAC > 0			
Subclinical hypothyroidism	0.55 (0.23; 1.30)	0.41 (0.16; 1.06)	0.46 (018; 1.18)
Only diabetes	1.27 (0.72; 2.25)	1.17 (0.63; 2.18)	0.99 (0.51; 1.91)
Both diseases	0.69 (0.19; 2.56)	1.32 (0.30;5.98)	0.89 (0.22; 3.59)
Linear regression models			
Log CAC + 1	β (95%CI)	β (95%CI)	β (95%CI)
Subclinical hypothyroidism	-0.11 (-0.80; 0.57)	0.36 (-0.34; 1.06)	0.39 (-0.34; 1.11)
Only diabetes	0.18 (-0.10; 0.46)*	0.17 (-0.10; 0.45)*	0.05 (-0.24; 0.34)*
Both diseases	-0.17 (-0.10; 0.46)	-0.23 (-0.68; 0.22)	-0.16 (-0.62; 0.29)
Logistic regression models			
CAC > 0	OR (95%CI)	OR (95%CI)	OR (95%CI)
Subclinical hypothyroidism	1.03 (0.66-1.63)	0.87 (0.53-1.43)	0.87 (0.57-1.33)
Only diabetes	2.68 (1.97-3.65)	1.71 (1.20-2.44)	1.23 (0.92-1.66)
Both diseases	1.67 (0.75-3.73)	0.67 (0.27-1.68)	1.12 (0.38-3.23)
Linear regression models			
Log CAC + 1	β (95%CI)	β (95%CI)	β (95%CI)
Subclinical hypothyroidism	0.21 (-0.01; 0.42)	0.21 (-0.006; 0.42)	0.07 (0.15; 0.28)
Only diabetes	0.26 (0.17; 0.36)*	0.27 (0.18; 0.36)*	0.17 (0.08; 0.26)*
Both diseases	0.15 (-0.66; 0.37)	0.04 (-0.07; 0.14)	-0.06; 0.14)

* P < 0.05; CAC = coronary artery calcium; Model 1 adjusted for age, sex, race and education attainment; Model 2 = Model 1 plus smoking, alcohol intake, and dyslipidemia.

In the stratified analysis, we found great heterogeneity among the groups. In the younger age strata, we found an OR = 7.16; 95%CI: 1.14-44.89. Although the interactions terms of diabetes and subclinical hypothyroidism were not statistically significant for logistic (P = 0.97) or linear models (P = 0.50) in stratified analysis, the wide confidence interval suggests an effect of the smaller sample size in each group compared to the main analysis in the entire sample (Table 2). In the analysis, considering the entire sample, we found no addictive or multiplicative effect in the association of diabetes and subclinical hypothyroidism with CAC. The interaction terms of diabetes and subclinical hypothyroidism are not statistically significant in logistic (P = 0.29) or linear models (P = 0.11).

In logistic models, there was an association between diabetes and CAC > 0 even after a multivariable adjustment for age, sex, education, smoking, hypertension, dyslipidemia, alcohol intake and physical activity (OR: 1.31; 95%CI: 1.05-1.63). However, we found no association among the participants with only subclinical hypothyroidism (OR: 0.94; 95%CI: 0.69-1.29) or both diseases (OR: 0.63; 95%CI: 0.32-1.23). In the linear models with the same multivariable adjustment, we also confirmed an association with diabetes with log (CAC+1) (β: 0.236; 95%CI: 0.163 to 0.403). We found no other significant associations considering only subclinical hypothyroidism, diabetes or both diseases.

Supplementary Table 1 describes the association of subclinical hypothyroidism diabetes and both diseases with CAC > 0 according to BMI < 30 kg/m^2^ and ≥ 30 kg/m^2^. Although in linear regression models participants with diabetes in both BMI categories were associated with log CAC+1, patients with both diseases were not. Interaction terms between diabetes and subclinical hypothyroidism for the association with CAC values in logistic and linear models were non-significant. Supplementary Table 2 shows the results of the analysis according to sex. Only diabetes was associated with CAC in linear models for men and women. The results remained non-significant.

**Supplementary Table 1:** Logistic models (Odds ratio [OR] and 95% Confidence Interval [95% CI]) and linear regression models (β coefficient [β] (95%CI) for the association of subclinical hypothyroidism, diabetes and both diseases with CAC >0 according to BMI < 30 kg/m^2^ or BMI ≥30 kg/m^2^ (obesity)

BMI < 30 kg/m^2^	Logistic regression models (OR, 95%CI)		
	Crude	Model 1	Model 2
**CAC>0**			
Only subclinical hypothyroidism	1.03 (0.75; 1.41)	0.87 (0.60; 1.24)	0.83 (0.57; 1.21)
Only diabetes	2.93 (2.32; 3.70)	1.68 (1.27; 2.22)	1.28 (0.95; 1.72)
Both diabetes and subclinical hypothyroidism	1.83 (0.83; 4.02)	1.24 (0.50; 3.06)	0.99 (0.38; 2.56)
**Log CAC+1**	**Linear regression models (β; 95% CI)**		
	**Crude**	**Model 1**	**Model 2**
Only subclinical hypothyroidism	0.30 (-0.009; 0.61)	0.32 (0.02; 0.61)	0.13 (-0.17; 0.43)
Only diabetes	0.49 (0.40; 0.58)*	0.41 (0.32; 0.50)	0.25 (0.16; 0.34)*
Both diabetes and subclinical hypothyroidism	0.01 (-0.10; 0.12)	-0.04 (-0.11; 0.10)	-0.02 (-0.12; 0.09)
**BMI ≥30 kg/m^2^**	**Logistic regression models (OR; 95% CI)**		
	**Crude**	**Model 1**	**Model 2**
**CAC>0**			
Only subclinical hypothyroidism	1.34 (0.76; 2.35)	1.08 (0.57; 2.06)	1.07 (0.55; 2.09)
Only diabetes	2.17 (1.58; 2.98)	1.37 (0.94; 2.00)	1.32 (0.89; 1.97)
Both diabetes and subclinical hypothyroidism	0.98 (0.41; 2.36)	0.65 (0.24; 1.79)	0.63 (0.22; 1.86)
	**Linear regression models (β 95% CI)**		
	**Crude**	**Model 1**	**Model 2**
**Log CAC + 1**			
Only subclinical hypothyroidism	0.02 (-0.31; 0.34)	0.02 (-0.31; 0.35)	-0.08 (-0.41; 0.26)
Only diabetes	0.37 (0.24; 0.50)*	0.31 (0.18; 0.44)*	0.22 (0.09; 0.35)*
Both diabetes and subclinical hypothyroidism	0.22 (0.002; 0.45)	0.20 (-0.02; 0.41)	0.14 (-0.07; 0.36)

* P < 0.05; CAC = coronary artery calcium; Model 1 adjusted for age, sex, race and education attainment; Model 2 = Model 1 plus smoking, alcohol intake, and dyslipidemia.

**Supplementary Table 2:** Odds ratio (95% Confidence Interval) of the association of subclinical thyroid disorders, diabetes and both diseases with coronary artery calcium CAC > 0 according to sex

	Logistic regression models (OR; 95% CI)		
	Crude	Model 1	Model 2
**Women**			
**CAC > 0**			
Only subclinical hypothyroidism	1.03 (0.66; 1.63)	0.87 (0.53; 1.43)	0.91 (0.54; 1.53)
Only diabetes	2.68 (1.97; 3.65)	1.71 (1.20; 2.44)	1.44 (0.98; 2.13)
Both diabetes and subclinical hypothyroidism	1.67 (0.75; 3.73)	0.67 (0.27; 1.68)	0.58 (0.22; 1.50)
**Log CAC + 1**	**Linear regression models (β, 95% CI)**		
	**Crude**	**Model 1**	**Model 2**
Only subclinical hypothyroidism	0.21 (-0.001; 0.42)	0,21 (-0,006; 0,42)	0.07 (-0.15; 0.28)
Only diabetes	0.28 (0.19; 0.36)*	0.27 (0.18; 0,36)*	0.17 (0.08; 0.26)*
Both diabetes and subclinical hypothyroidism	0.04 (-0.07; 0.14)	0.04 (-0,07; 0.14)	0.04 (-0,06; 0,14)
	**Logistic regression models (OR, 95% CI)**		
	**Crude**	**Model 1**	**Model 2**
**Men**			
**CAC > 0**			
Only subclinical hypothyroidism	1.11 (0.78; 1.59)	0.95 (0.63; 1.42)	0.87 (0.57; 1.33)
Only diabetes	2.26 (1.77; 2.88)*	1.59 (1.21; 2.10)*	1.23 (0.92; 1.66)*
Both diabetes and subclinical hypothyroidism	1.49 (0.61; 3.61)	1.40 (0.52; 3.75)	1.12 (0.38; 3.25)
**Log CAC + 1**	**Linear regression models (β, 95% CI)**		
	**Crude**	**Model 1**	**Model 2**
Only subclinical hypothyroidism	0.16 (-0.26; 0.58)	0.14 (-0.34; 0.37)	0.08 (-0.34; 0.50)
Only diabetes	0.47 (0.36; 0.58)*	0.45 (0.34; 0.56)*	0.28 (0.17; 0.39)*
Both diabetes and subclinical hypothyroidism	0.07 (-0.09; 0.24)	0.03 (-0.13; 0.20)	(-0.16; 0.16)

* P < 0.05; CAC = coronary artery calcium; Model 1 adjusted for age, sex, race and education attainment; Model 2 = Model 1 plus smoking, alcohol intake, and dyslipidemia.

## DISCUSSION

In the main analysis, we found no association of diabetes and subclinical hypothyroidism with CAC. However, in stratified analysis according to age, we found an association of patients with subclinical hypothyroidism and diabetes with CAC in the younger age strata group. Given the magnitude of point OR estimates, there may be some kind of interaction in the multiplicative and additive scales even though the interaction terms were not statistically significant in the youngest age stratum because of the small sample size in each group in the stratified analysis. We also found an association of logistic and linear regression models of diabetes with CAC.

Some points may be highlighted to explain our results. The number of participants with both diseases was only 55, which may not be enough to detect a positive association, especially if the strength of the association was not very high, which is the case in this analysis. The mean age of participants in the ELSA-Brasil at baseline examination was around 50 years. The prevalence of diabetes and subclinical hypothyroidism increases with age, as well as the presence of subclinical atherosclerosis. Therefore, the sample may be too young to test our hypothesis considering all age strata together. Some imbalance in the distribution of diabetes according to sex shows more male patients with diabetes whereas subclinical hypothyroidism is more frequent in women. The possible interaction effects of having subclinical hypothyroidism and diabetes are also likely not homogeneous considering their different outcomes.

We hypothesized that the association of diabetes with subclinical hypothyroidism in the same patients would result in more subclinical atherosclerosis, reflected by a stronger association with CAC in this subgroup compared only to patients with diabetes. However, our results are not as clear as in a previous analysis of the ELSA-Brasil study, which revealed a clear additive effect between diabetes and subclinical hypothyroidism impacting the lower cardiac autonomic control in the subgroup of participants with both diseases compared to other subgroups. In addition, in the same analysis, a borderline-significant interaction occurred between diabetes and subclinical hypothyroidism on heart rate compared to patients with only diabetes (37).

In relation to the negative results in the main analysis, it is possible to say that when we analyze together all age strata, some differences related to age disappear and may be hidden, showing negative results. In addition, although the adoption of a single cutoff value for SCH classification for all ages enhances comparability, it may have led to some misclassification, which may have contributed to our negative results when we analyzed data from older participants.

The plausibility of our hypothesis of an association of diabetes and subclinical hypothyroidism may be supported by scientific evidence. Some studies, including one meta-analysis (7), have shown that subclinical hypothyroidism was associated with a higher prevalence of overall risk of diabetes and its complications (7–9,11). However, other studies did not confirm these associations. Sharma and cols. (2020) reported that patients with diabetes and subclinical hypothyroidism have similar glycemic control as patients with only diabetes (38), and Mehalingam and cols. (2020) reported that patients with both diseases did not present more severe complications of diabetes compared to patients with only diabetes (39). In addition, overt and subclinical hypothyroidism are associated with higher peripheral glucose levels (40,41), decreased glucose use (42) and insulin resistance (43,44). However, there is also evidence that overt and subclinical hyperthyroidism have been associated with increased hepatic gluconeogenesis (45), increased insulin clearance (46) and resistance (47), providing a plausible justification for a U-shaped curve. The original idea in the present analysis was to evaluate both subclinical thyroid diseases. However, the number of patients with diabetes and subclinical hyperthyroidism was very small in the sample (N = 3).

We also found an association with diabetes and CAC in logistic and linear models in the younger age strata. We conducted two sensitivity analyses according to BMI categories and sex. In the group of BMI < 30 kg/m^2^ and in the group ≥ 30 kg/m^2^, diabetes was associated with CAC > 0. In the analysis according to sex, we also found an association between diabetes and CAC in linear regression models for men and women. However, we found no association considering the subgroup with both diseases, and we detected no significant interaction between diabetes and subclinical hypothyroidism.

Some studies have shown an association of low TSH levels with CAC (20,21). In fact, Peixoto de Miranda and cols. (2017) found that lower and higher TSH levels were associated with CAC showing a U-shaped curve in women but not in men (20,21). The association between diabetes and atherosclerosis as we report it here is well known (48–50). The CAC score in patients with diabetes help in risk stratification of patients with diabetes and intermediary risk (50,51). Coronary artery calcium is also a predictor of cardiovascular events in asymptomatic patients with type 2 diabetes. Contrasting with these positive results for subclinical hypothyroidism and even for diabetes (18,19,51), studies showed no association with subclinical atherosclerosis measured by CAC. Researchers conducted few studies to evaluate an association of subclinical hypothyroidism, diabetes or metabolic syndrome with CAC in high-risk subgroups. Posadas-Romero and cols. (2014) reported an association of subclinical hypothyroidism, metabolic syndrome and its components with subclinical atherosclerosis measured by CAC and fat-liver disease (22). In addition, a cross-sectional study in Brazil reported a strong positive association between CAC >100 and subclinical hypothyroidism in older men with a Framingham risk score ≥ 10% and having diabetes as one of the cardiovascular risk factors evaluated (23). Recently, patients with higher TSH levels and diabetes were associated with high CAC in hemodialysis (24). It is important to note that all these studies showed possible associations among subclinical hypothyroidism, diabetes and CAC in high-risk groups. One challenge is to find studies that include information about subclinical hypothyroidism, diabetes and CAC because most studies to evaluate this association included only euthyroid participants.

Our study must, however, be read considering the limitations and context of its cross-sectional design, which does not permit the evaluation of causal associations. The number of cases of diabetes and subclinical hypothyroidism is not so high, limiting our power to conduct the sensitivity analysis. Another limitation is the small number of high TSH values in the sample. The CAC score was measured at baseline when the participants were younger, with a mean age around 50 years, with a small number of participants with higher CAC values. In addition, some kind of misclassification in the diagnosis of subclinical hypothyroidism that was defined by the entire sample and not according to age may have contributed to our negative results. The analysis also has some strength. The ELSA-Brasil used centralized protocols to train the research team under strict quality control. The diagnosis of diabetes was very comprehensive and included previous medical history of diabetes, use of medication to treat diabetes, fasting plasma glucose and an oral glucose tolerant test as well as HbA1c. The study also describes a highly admixed sample from a middle-income country with different characteristics compared to samples analyzed in previous studies.

In conclusion, our results showed no association between the group with both diseases and CAC in the main analysis. However, the findings showed a great heterogeneity in stratified analysis according to age, with a strong association in the younger age strata. Although we found no significant interaction factors, the smaller sample size in stratified analysis may influence the negative findings.

## References

[1] International Diabetes Federation (2019). IDF Diabetes Atlas.

[2] Tönnies T, Rathmann W, Hoyer A, Brinks R, Kuss O (2021). Quantifying the underestimation of projected global diabetes prevalence by the International Diabetes Federation (IDF) Diabetes Atlas. BMJ Open Diabetes Res Care.

[3] Chaker L, Bianco AC, Jonklaas J, Peeters RP (2017). Hypothyroidism. Lancet.

[4] De Leo S, Lee SY, Braverman LE (2016). Hyperthyroidism. Lancet.

[5] Cooper DS, Biondi B (2012). Subclinical thyroid disease. Lancet.

[6] Biondi B, Kahaly GJ, Robertson RP (2019). Thyroid Dysfunction and Diabetes Mellitus: Two Closely Associated Disorders. Endocr Rev.

[7] Han C, He X, Xia X, Li Y, Shi X, Shan Z (2015). Subclinical Hypothyroidism and Type 2 Diabetes: A Systematic Review and Meta-Analysis. PLoS One.

[8] Mansournia N, Riyahi S, Tofangchiha S, Mansournia MA, Riahi M, Heidari Z (2017). Subclinical hypothyroidism and diabetic nephropathy in Iranian patients with type 2 diabetes. J Endocrinol Invest.

[9] Jia F, Tian J, Deng F, Yang G, Long M, Cheng W (2015). Subclinical hypothyroidism and the associations with macrovascular complications and chronic kidney disease in patients with Type 2 diabetes. Diabet Med.

[10] Xie J, Wang X, Zhang Y, Li H, Xu Y, Zheng D (2019). The longitudinal effect of subclinical hypothyroidism on urine microalbumin-to-urine creatinine ratio in patients with type 2 diabetes mellitus. BMC Endocr Disord.

[11] Reddy N, Pradeep TVS, Tirupati S, Sarathi V, Kumar D (2020). Thyroid dysfunction and its association with microvascular complications in patients with type 2 diabetes mellitus in south India. Diabetes Metab Syndr.

[12] Allam MA, Nassar YA, Shabana HS, Mostafa S, Khalil F, Zidan H (2021). Prevalence and Clinical Significance of Subclinical Hypothyroidism in Diabetic Peripheral Neuropathy. Int J Gen Med.

[13] Fleiner HF, Bjøro T, Midthjell K, Grill V, Åsvold BO (2016). Prevalence of Thyroid Dysfunction in Autoimmune and Type 2 Diabetes: The Population-Based HUNT Study in Norway. J Clin Endocrinol Metab.

[14] Ishay A, Chertok-Shaham I, Lavi I, Luboshitzky R (2009). Prevalence of subclinical hypothyroidism in women with type 2 diabetes. Med Sci Monit.

[15] Gopinath B, Wang JJ, Kifley A, Wall JR, Leeder SR, Mitchell P (2008). Type 2 diabetes does not predict incident thyroid dysfunction in the elderly. Diabetes Res Clin Pract.

[16] Silverman MG, Blaha MJ, Krumholz HM, Budoff MJ, Blankstein R, Sibley CT (2014). Impact of coronary artery calcium on coronary heart disease events in individuals at the extremes of traditional risk factor burden: the Multi-Ethnic Study of Atherosclerosis. Eur Heart J.

[17] Blankstein R, Budoff MJ, Shaw LJ, Goff DC, Polak JF, Lima J (2011). Predictors of coronary heart disease events among asymptomatic persons with low-density lipoprotein cholesterol MESA (Multi-Ethnic Study of Atherosclerosis). J Am Coll Cardiol.

[18] Mamudu HM, Alamian A, Paul T, Subedi P, Wang L, Jones A (2018). Diabetes, subclinical atherosclerosis and multiple cardiovascular risk factors in hard-to-reach asymptomatic patients. Diab Vasc Dis Res.

[19] Jeevarethinam A, Venuraju S, Dumo A, Ruano S, Mehta VS, Rosenthal M (2017). Relationship between carotid atherosclerosis and coronary artery calcification in asymptomatic diabetic patients: A prospective multicenter study. Clin Cardiol.

[20] Peixoto de Miranda ÉJF, Bittencourt MS, Staniak HL, Pereira AC, Foppa M, Santos IS (2017). Thyrotrophin levels and coronary artery calcification: Cross-sectional results of the Brazilian Longitudinal Study of Adult Health (ELSA-Brasil). Clin Endocrinol (Oxf).

[21] Zhang Y, Kim BK, Chang Y, Ryu S, Cho J, Lee WY (2014). Thyroid hormones and coronary artery calcification in euthyroid men and women. Arterioscler Thromb Vasc Biol.

[22] Posadas-Romero C, Rosalinda Posadas-Sánchez E, Acuña-Valerio J, Juárez-Rojas JG, Kimura-Hayama E, Medina-Urrutia A (2014). Fatty liver largely explains associations of subclinical hypothyroidism with insulin resistance, metabolic syndrome, and subclinical coronary atherosclerosis. Eur J Endocrinol.

[23] Silva N, Santos O, Morais F, Gottlieb I, Hadlich M, Rothstein MT (2022). Subclinical hypothyroidism represents an additional risk factor for coronary artery calcification, especially in subjects with intermediate and high cardiovascular risk scores. Cardiorenal Med.

[24] Rhee CM, Budoff M, Brent G, You AS, Stenvinkel P, Novoa A (2014). Serum Thyrotropin Elevation and Coronary Artery Calcification in Hemodialysis Patients. Eur J Endocrinol.

[25] Aquino EM, Barreto SM, Bensenor IM, Carvalho MS, Chor D, Duncan BB (2012). Brazilian Longitudinal Study of Adult Health (ELSA-Brasil): objectives and design. Am J Epidemiol.

[26] Schmidt MI, Duncan BB, Mill JG, Lotufo PA, Chor D, Barreto SM (2015). Cohort Profile: Longitudinal Study of Adult Health (ELSA-Brasil). Int J Epidemiol.

[27] Bensenor IM, Griep RH, Pinto KA, Faria CP, Felisbino-Mendes M, Caetano EI (2013). Rotinas de organização de exames e entrevistas no centro de investigação ELSA-Brasil. Rev Saude Publica.

[28] Schmidt MI, Hoffmann JF, Diniz MDFS, Lotufo PA, Griep RH, Bensenor IM (2014). High prevalence of diabetes and intermediate hyperglycemia - The Brazilian Longitudinal Study of Adult Health (ELSA-Brasil). Diabetol Metab Syndr.

[29] Fedeli LG, Vidigal PG, Leite CM, Castilhos CD, Pimentel RA, Maniero VC (2013). Logística de coleta e transporte de material biológico e organização do laboratório central no ELSA-Brasil. Rev Saude Publica.

[30] Olmos RD, Figueiredo RC, Aquino EM, Lotufo PA, Bensenor IM (2015). Gender, race and socioeconomic influence on diagnosis and treatment of thyroid disorders in the Brazilian Longitudinal Study of Adult Health (ELSA-Brasil). Braz J Med Biol Res.

[31] Lai EC, Yang YH, Lin SJ, Hsieh CY (2013). Use of antiepileptic drugs and risk of hypothyroidism. Pharmacoepidemiol Drug Saf.

[32] Dong BJ (2000). How medications affect thyroid function. West J Med.

[33] Bensenor IM, Goulart AC, Santos IS, Bittencourt MS, Pereira AC, Santos RD (2016). Association between a healthy cardiovascular risk factor profile and coronary artery calcium score: Results from the Brazilian Longitudinal Study of Adult Health (ELSA-Brasil). Am Heart J.

[34] Pereira AC, Gomez LM, Bittencourt MS, Staniak HL, Sharovsky R, Foppa M (2016). Age, Gender, and Race-Based Coronary Artery Calcium Score Percentiles in the Brazilian Longitudinal Study of Adult Health (ELSA-Brasil). Clin Cardiol.

[35] Agatston AS, Janowitz WR, Hildner FJ, Zusmer NR, Viamonte M, Detrano R (1990). Quantification of coronary artery calcium using ultrafast computed tomography. J Am Coll Cardiol.

[36] Matsudo S, Araújo T, Matsudo V, Andrade D, Andrade E, Oliveira LC (2012). Questionário internacional de atividade física (IPAQ): Estudo de validade e reprodutibilidade no Brasil. Rev Bras Ativ Fís Saúde.

[37] Hoshi RA, Santos IS, Dantas EM, Andreão RV, Mill JG, Duncan BB (2020). Diabetes and subclinical hypothyroidism on heart rate variability. Eur J Clin Invest.

[38] Sharma P, Sinha R, Prasad A, Mitra JK (2020). Lack of Association between Poor Glycemic Control in T2DM and Subclinical Hypothyroidism. J Thyroid Res.

[39] Mehalingam V, Sahoo J, Bobby Z, Vinod KV (2020). Thyroid dysfunction in patients with type 2 diabetes mellitus and its association with diabetic complications. J Family Med Prim Care.

[40] Cettour-Rose P, Theander-Carrillo C, Asensio C, Klein M, Visser TJ, Burger AG (2005). Hypothyroidism in rats decreases peripheral glucose utilisation, a defect partially corrected by central leptin infusion. Diabetologia.

[41] Waring AC, Rodondi N, Harrison S, Kanaya AM, Simonsick EM, Miljkovic I (2012). Health, Ageing, and Body Composition (Health ABC) Study. Thyroid function and prevalent and incident metabolic syndrome in older adults: the Health, Ageing and Body Composition Study. Clin Endocrinol (Oxf).

[42] Brenta G, Celi FS, Pisarev M, Schnitman M, Sinay I, Arias P (2009). Acute thyroid hormone withdrawal in athyreotic patients results in a state of insulin resistance. Thyroid.

[43] Maratou E, Hadjidakis DJ, Kollias A, Tsegka K, Peppa M, Alevizaki M (2009). Studies of insulin resistance in patients with clinical and subclinical hypothyroidism. Eur J Endocrinol.

[44] Foss MC, Paccola GM, Saad MJ, Pimenta WP, Piccinato CE, Iazigi N (1990). Peripheral glucose metabolism in human hyperthyroidism. J Clin Endocrinol Metab.

[45] Mitrou P, Raptis AS, Dimitriadis G (2010). Insulin action in hyperthyroidism: a focus on muscle and adipose tissue. Endocr Rev.

[46] Randin JP, Tappy L, Scazziga B, Jequier E, Felber JP (1986). Insulin sensitivity and exogenous insulin clearance in Graves’ disease. Measurement by the glucose clamp technique and continuous indirect calorimetry. Diabetes.

[47] Maratou E, Hadjidakis DJ, Peppa M, Alevizaki M, Tsegka K, Lambadiari V (2010). Studies of insulin resistance in patients with clinical and subclinical hyperthyroidism. Eur J Endocrinol.

[48] Lei MH, Wu YL, Chung SL, Chen CC, Chen WC, Hsu YC (2021). Coronary Artery Calcium Score Predicts Long-Term Cardiovascular Outcomes in Asymptomatic Patients with Type 2 Diabetes. J Atheroscler Thromb.

[49] Young LH, Wackers FJ, Chyun DA, Davey JA, Barrett EJ, Taillefer R, Heller GV, Iskandrian AE, Wittlin SD, Filipchuk N, Ratner RE, Inzucchi SE, DIAD Investigators (2009). Cardiac outcomes after screening for asymptomatic coronary artery disease in patients with type 2 diabetes: the DIAD study: a randomized controlled trial. JAMA.

[50] Malik S, Budoff MJ, Katz R, Blumenthal RS, Bertoni AG, Nasir K (2011). Impact of subclinical atherosclerosis on cardiovascular disease events in individuals with metabolic syndrome and diabetes: the multi-ethnic study of atherosclerosis. Diabetes Care.

[51] Nicoll R, Zhao Y, Ibrahimi P, Olivecrona G, Henein M (2016). Diabetes and Hypertension Consistently Predict the Presence and Extent of Coronary Artery Calcification in Symptomatic Patients: A Systematic Review and Meta-Analysis. Int J Mol Sci.

